# Bactericidal activities of the cationic steroid CSA-13 and the cathelicidin peptide LL-37 against *Helicobacter pylori *in simulated gastric juice

**DOI:** 10.1186/1471-2180-9-187

**Published:** 2009-09-03

**Authors:** Katarzyna Leszczyńska, Andrzej Namiot, David E Fein, Qi Wen, Zbigniew Namiot, Paul B Savage, Scott Diamond, Paul A Janmey, Robert Bucki

**Affiliations:** 1 Department of Diagnostic Microbiology, Medical University of Bialystok, 15-230 Bialystok, Poland; 2 Department of Anatomy, Medical University of Bialystok, 15-230 Bialystok, Poland; 3 Penn Center for Molecular Discovery, Institute for Medicine and Engineering, Department of Chemical and Biomolecular Engineering, University of Pennsylvania, Philadelphia, PA 19104, USA; 4 Department of Physiology and the Institute for Medicine and Engineering, University of Pennsylvania, 1010 Vagelos Research Laboratories, 3340 Smith Walk, Philadelphia, PA, 19104 USA; 5 Department of Physiology, Medical University of Bialystok, 15-230 Bialystok, Poland; 6 Department of Chemistry and Biochemistry, Brigham Young University, C-I00 BNSN, Provo, UT 84602, USA

## Abstract

**Background:**

The worldwide appearance of drug-resistant strains of *H. pylori *motivates a search for new agents with therapeutic potential against this family of bacteria that colonizes the stomach, and is associated with adenocarcinoma development. This study was designed to assess *in vitro *the anti-*H. pylori *potential of cathelicidin LL-37 peptide, which is naturally present in gastric juice, its optimized synthetic analog WLBU2, and the non-peptide antibacterial agent ceragenin CSA-13.

**Results:**

In agreement with previous studies, increased expression of hCAP-18/LL-37 was observed in gastric mucosa obtained from *H. pylori *infected subjects. MBC (minimum bactericidal concentration) values determined in nutrient-containing media range from 100-800 μg/ml for LL-37, 17.8-142 μg/ml for WLBU2 and 0.275-8.9 μg/ml for ceragenin CSA-13. These data indicate substantial, but widely differing antibacterial activities against clinical isolates of *H. pylori*. After incubation in simulated gastric juice (low pH with presence of pepsin) CSA-13, but not LL-37 or WLBU2, retained antibacterial activity. Compared to LL-37 and WLBU2 peptides, CSA-13 activity was also more resistant to inhibition by isolated host gastric mucins.

**Conclusion:**

These data indicate that cholic acid-based antimicrobial agents such as CSA-13 resist proteolytic degradation and inhibition by mucin and have potential for treatment of *H. pylori *infections, including those caused by the clarithromycin and/or metronidazole-resistant strains.

## Background

*Helicobacter pylori *is carried by more than half of the world's adult population [1]. It can chronically colonize the human gastric mucosa, where it is found in the mucus layer and is adhered to epithelial cells [2]. Although most infected subjects remain asymptomatic, infection with *H. pylori *can promote severe gastritis [3] and significantly increase the risk of gastric malignancies [4,5]. In some epidemiological studies, *H. pylori *eradication was shown to be effective in gastric cancer prevention [6,7]. Additionally, *H. pylori *eradication was found to decrease the incidence and the severity of lesions with carcinogenic potential in animal models [8,9]. Natural mechanisms that protect the host from *H. pylori *infections depend on the function of the innate defense system in which antibacterial peptides such as cathelicidin LL-37 [10,11] and O-glycans in gastric mucin [12] play a key role.

LL-37 is a proteolytically processed peptide derived from the C-terminal domain of human cathelicidin (hCAP-18/LL-37) that is constitutively released to the extracellular space by phagocytic granulocytes and epithelial cells [13]. Functions ascribed to LL-37 include prevention of bacterial growth [14], neutralization of bacterial wall molecule bioactivity [15], and activation of host cells by binding specific cell membrane receptors [16-18]. *H. pylori *upregulates the production of LL-37/hCAP18 by the gastric epithelium, suggesting that cathelicidin or its derivative LL-37 contributes to determining the balance between host mucosal defense and *H. pylori *survival mechanisms that govern chronic infection with this gastric pathogen [10,11].

Cationic antibacterial peptides (CAPs) including LL-37 have been extensively investigated as a potential source of new antibacterial molecules. The engineered WLBU2 peptide whose residues are arranged to form an amphipathic helical structure with optimal charge and hydrophobic density, overcomes some limitations of natural LL-37 such as sensitivity to Mg^2+ ^or Ca^2+ ^and inactivation by blood serum [19]. Therefore WLBU2 could treat infections where LL-37 is ineffective. In order to generate molecules able to mimic CAPs' ability to compromise bacterial membrane integrity, non-peptide ceragenins with cationic, facially amphiphilic structures characteristic of most antimicrobial peptides were developed. Ceragenins such as CSA-13 reproduce the required CAP morphology using a bile-acid scaffolding and appended amine groups [20]. They are bactericidal against both Gram-positive and Gram-negative organisms, including drug-resistant bacteria such as clinically relevant methicillin-resistant *Staphylococcus aureus *(MRSA), and a previous susceptibility study demonstrated that CSA-13 has a MIC_50_/MBC_50 _ratio of 1 [21,22]. In this study we compare the bactericidal potency of LL-37, WLBU2 and CSA-13 against clinical isolates of *H. pylori*. The results suggest that cholic acid-based mimics of antimicrobial peptide such as CSA-13 have potential for treatment of *H. pylori *infection, including those caused by the clarithromycin and/or metronidazole-resistant strains.

## Results

### Immunohistochemical probing of human gastric mucosa sections with anti-hCAP-18/LL-37 antibody

**Figure 1:**
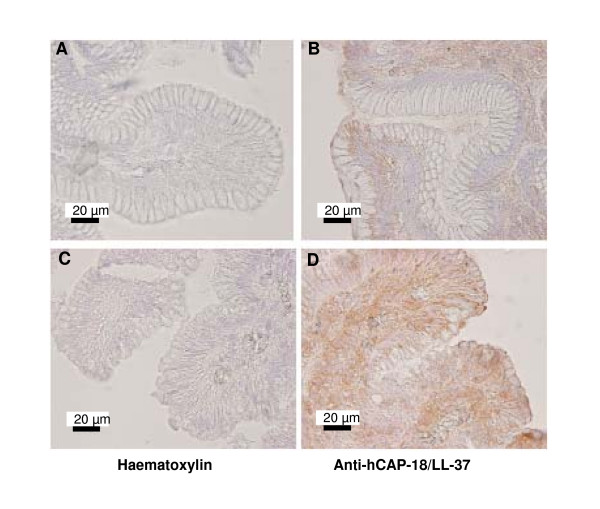
**Presence of hCAP-18/LL-37 peptide in mucosal biopsies from the human stomach detected using immunohistochemical analysis with monoclonal antibodies to human CAP-18/LL-37**. Samples A/B and C/D represent the specimens obtained from non-infected and *H. pylori *infected subjects respectively. Data shown are representative of five experiments.

Microscopic images of mucosal biopsies after immunohistochemical evaluation with anti-hCAP-18/LL-37 antibody are shown in Figure 1. The DAB-positive staining indicates the presence of the LL-37 peptide and/or its parent protein hCAP-18. High intensity DAB staining (indicated by brown color) at the mucus-producing epithelial cells and fundic glands indicates high accumulation of hCAP-18/LL-37 peptide most likely driven by LL-37 specific interaction with mucin, which was reported in previous studies [23,24]. The distribution of hCAP-18/LL-37 in the more differentiated epithelial cell population of the gastric mucosa differs from that found for human β-defensin 2 [10] or lysozyme [25] but is similar to that observed in the colon [26]. Gastric mucosal biopsies from patients infected with *H. pylori *show higher intensity of DAB staining compared to those obtained from non-infected subjects. According to previous reports, this result indicates a host defense response to *H. pylori *[11], which is partly based on increased expression of hCAP-18/LL-37 by gastric epithelial cells.

### Bactericidal activity of LL-37, WLBU-2 peptides and ceragenin CSA-13 against different strains of *H. pylori*

**Figure 2:**
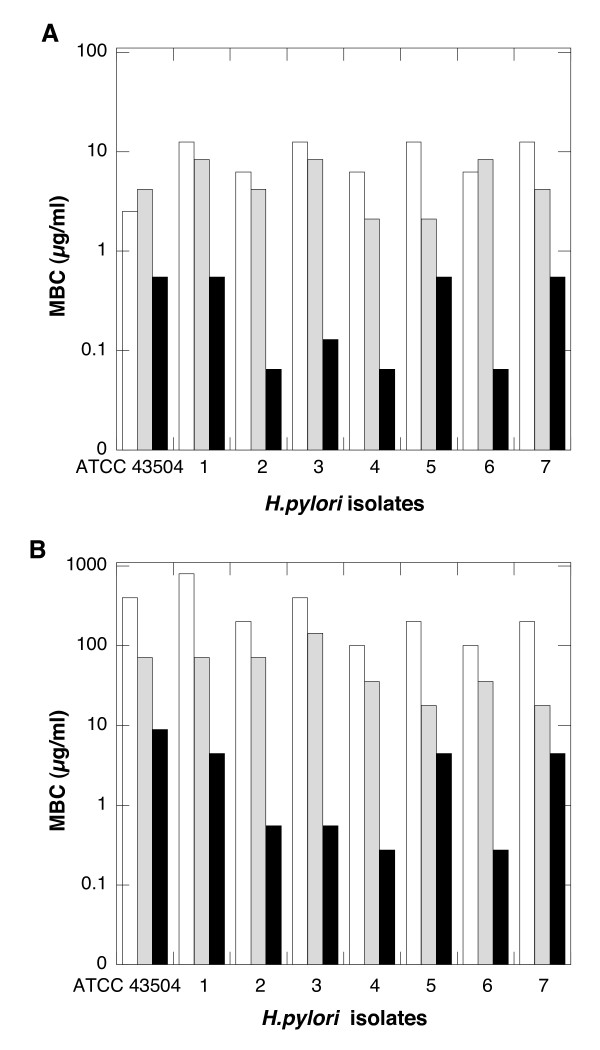
**Bactericidal activity against *H. pylori***. Minimum bactericidal concentration (MBC) of LL-37 (white column), WLBU2 (gray column) and CSA-13 (black column) against *H. pylori *(ATCC 43504 strain and seven clinical isolates obtained from mucosal samples from different subjects) evaluated in HEPES (panel A) or Brucella Broth Bulion (panel B). MBC indicates concentrations at which compounds completely eradicate an inoculum of *H. pylori*.

To identify resistant strains, clinical isolates of *H. pylori *were subjected to MIC evaluation (Table 1) with several antibiotics currently used in clinical treatment of *H. pylori *infection. Among seven tested isolates obtained from different subjects, strain 4 was resistant to metronidazole and strains 5, 6, 7 were resistant to both metronidazole and clarithromycin. All isolates were susceptible to amoxicillin and tetracycline. Consistent with previous reports on the effects of hBD-1, h-BD-2 and LL-37 peptides against *H. pylori *[10,11] all isolated strains of *H. pylori *were killed after 6 hours incubation with LL-37, WLBU2 and CSA-13 with average MBC (μg/ml) values 8.9 ± 4.03; 5.23 ± 2.7 and 0.31 ± 0.25 when MBC was evaluated in HEPES buffer, or 300 ± 232, 53 ± 41 and 2.98 ± 3.11 when MBC was evaluated in Brucella Broth Bullion respectively (Figure 2). Evaluation of MBC values in HEPES buffer with addition of 2 mM MgCl_2 _for *H. pylori *ATCC 43504 revealed an eight fold increase for LL-37, and a four fold increase for both WLBU2 and CSA-13 (data not show).

**Table 1:** Evaluation of sensitivity of clinical strains of *H. pylori *to antibiotics.

***H. pylori *strains**	**Antibiotics**			
	
	AMX	CLR	TET	Metronidazole
ATCC 43504	0.016	0.094	0.25	64.0 ^®^
1	0.094	0.125	0.75	0.19
2	<0.016	0.19	0.125	0.094
3	0.016	0.25	3.0	0.5
4	0.032	0.047	2.0	32.0 ^®^
5	0.25	64.0 ^®^	1.0	96.0 ^®^
6	0.032	1.5 ^®^	1.5	32.0 ^®^
7	0.047	1.5 ^®^	2.0	48.0 ^®^

MIC values (μg/ml) (AMX-amoxicillin, CLR-clarithromycin, TET-tetracycline)

### Antibacterial activity of LL-37, WLBU2 and CSA-13 after pre-incubation at low pH with pepsin or mucin

**Figure 3:**
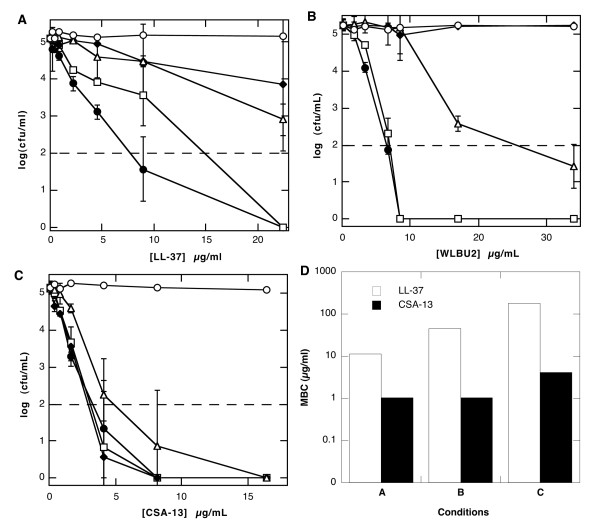
**Antibacterial activity against *E. coli *MG1655 and *H. pylori *strain ATCC 43504**. Antibacterial activity of LL-37 (panel A), WLBU2 (panel B) and CSA-13 (panel C) against *E. coli *MG1655 after pre-incubation (3 h at 37°C) in PBS (open circles), simulated gastric juice at pH ~1.5 (squares), simulated gastric juice with pepsin (diamonds), simulated gastric juice with mucin (triangles) and PBS with human bile (10%) obtained from the gallbladder (filled circle). Data shown are means ± SD of three to four experiments. MBC of LL-37 (white column) and CSA-13 (black column) (panel D) against *H. pylori *(ATCC 43504) after pre-incubation (1 h at 37°C) in simulated gastric juice at pH ~1.5 (A), simulated gastric juice with pepsin (B) and in presence of mucin (C)

In addition to known inhibition of CAPs antibacterial activity by divalent cations such as Mg^2+ ^and Ca^2+^, the proteolytic activity of pepsin may also compromise CAPs function in the gastric juice environment with the presence of mucins, and low pH. To address this possibility we evaluated the antibacterial activity against *Escherichia coli *MG1655 after 3 hours pre-incubation of LL-37, WLBU2 and CSA-13 in simulated gastric juice in comparison to activity after their pre-incubation in PBS at pH 7.4. Before conducting the killing assay, the pH of samples with low pH and low pH/pepsin was adjusted to 7.4. The antibacterial activity of LL-37 and WLBU2 peptides against *E. coli *MG1655 was not significantly changed after pre-incubation at pH ~1.5, but was lost after pre-incubation at pH ~1.5 in the presence of pepsin (Figure 3A and 3B). In contrast, the antibacterial activity of CSA-13 was unchanged by pre-incubation at pH ~1.5 with or without pepsin (Figure 3C). On the other hand, bactericidal activities of all components were compromised to various extents when tested using a bacterial killing assay in the presence of purified gastric mucin. In close agreement with results obtained from this *E. coli *MG1655 study, MBC values of LL-37 peptide evaluated after 1H pre-incubation with buffer at low pH containing pepsin or mucin was increased but those of CSA-13 were nearly unchanged (Figure 3D). All evaluated agents lost antibacterial activity in PBS supplemented with 10% human bile (a concentration that does not interfere with *E. coli *MG1655 growth - data not shown). This result suggests that physico-chemical properties of antibacterial molecules promote their insertion in bile lipoprotein, thereby limiting their interaction with the bacterial wall. There has been no study to evaluate antibacterial activity of CAPs in duodenal juice, but these results indicate that bile reflux into the stomach may interfere with CAPs activity.

### Analytical characterization of LL-37 and CSA-13 after incubation with pepsin

**Figure 4:**
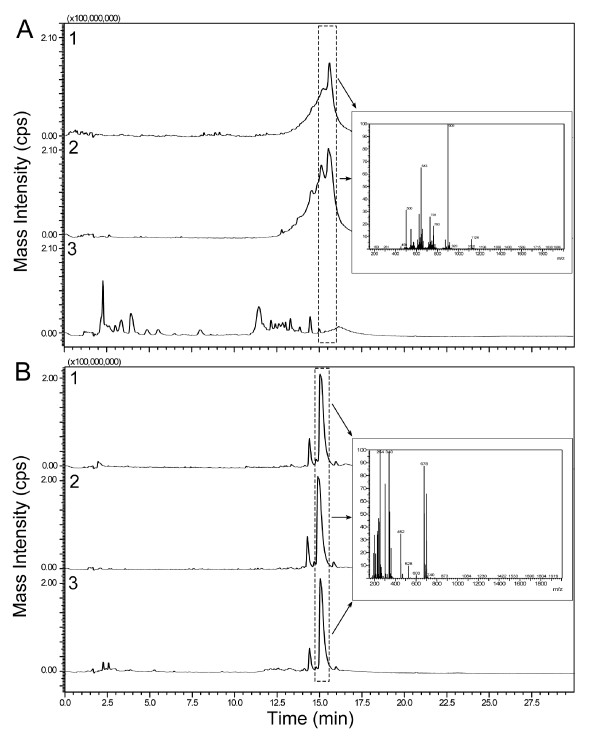
**Mass spectrometry analysis**. Mass spectrometry analysis of LL-37 (panel A) and CSA-13 (panel B) in PBS (curve 1) low pH buffer (curve 2) and low pH buffer with presence of pepsin (curve 3). The total ion chromatogram (TIC) is presented for each sample condition with an inset mass-to-charge (m/z) spectra showing intensity for the boxed TIC peaks. The molecular weight of intact LL-37 is 4494, which can be observed with multiple charges (m/z = 4 MW = 1124, m/z = 5 MW = 900, etc) in positive ion mode. The molecular weight of CSA13 is 678, which can be observed directly and with multiple charges. Data from one experiment are shown.

Mass spectrometry analysis (Figure 4) reveals that three hours incubation with pepsin results in extensive degradation of LL-37. However, at low pH, pepsin digestion is highly specific and LL-37 peptide cleavage is limited to the site with hydrophobic amino acids. Potential cleavage sites predicted by PeptideCutter characterization software http://kr.expasy.org/tools/peptidecutter/, suggest that LL-37 digestion with pepsin in our experimental conditions should release 11 products, including 3 shorter peptides (RKSKEKIGKE, FKRIVQRIKD and LVPRTES). These predictions are consistent with mass spectral analysis, which does not show the presence of any intact LL-37 remaining following incubation with pepsin at low pH, but does reveal the emergence of multiple new peaks with different retention times. The remaining antibacterial activity of LL-37 following treatment with pepsin (Figure 3A and 3D) in the killing assays likely represents the residual activity of these LL-37 fragments. Contrary to the observed degradation of LL-37, CSA-13 analytical characterization was not changed after incubation with pepsin at low pH.

### Toxicity of LL-37, WLBU2 and CSA-13 against RBC and human adenocarcinoma cells

**Figure 5:**
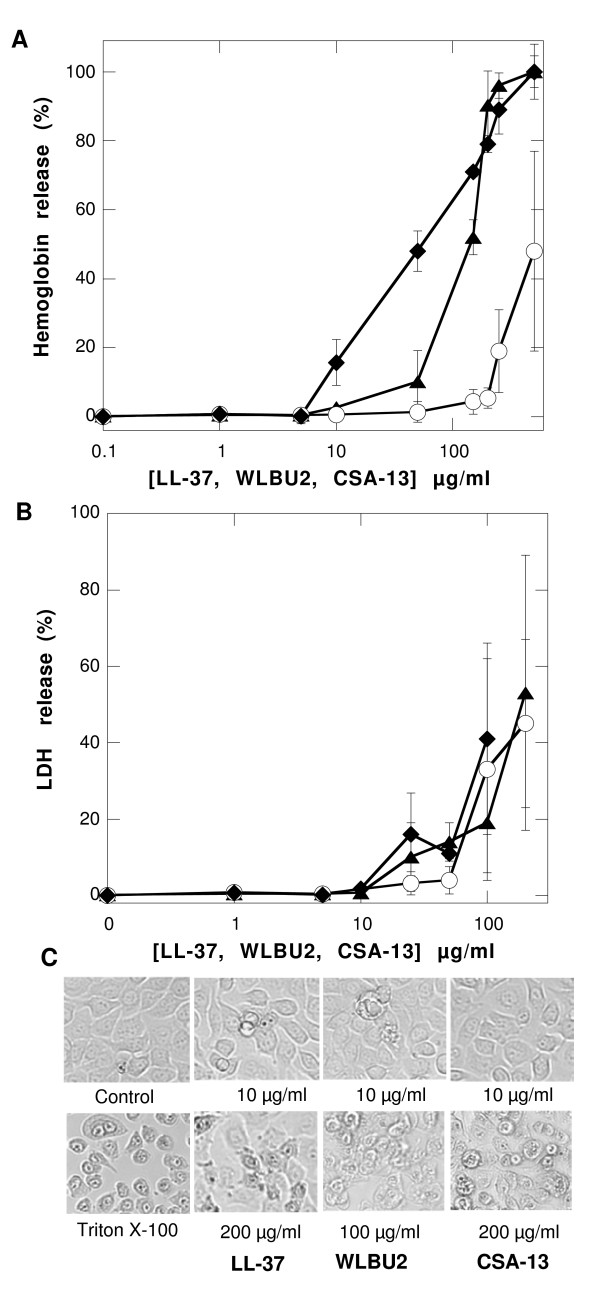
**Evaluation of cell toxicity**. Hemoglobin and LDH release from human red blood cells and human gastric adenocarcinoma cells (panel A and B respectively) after addition of LL-37 (circles), WLBU2 (diamonds), and CSA-13 (triangles), followed by incubation for 1 h at 37°C. Data shown are means ± SD of three experiments. Morphology of human gastric adenocarcinoma cells before (control) and after LL-37, WLBU2 and CSA-13 treatment was evaluated by phase-contrast microscopy (panel C). Data from one representative experiment are shown. Two other experiments revealed similar results.

Non-specific insertion of antibacterial peptides and their mimics into host cell membranes can cause toxicity. Host cell membrane permeabilization can be measured by the release of proteins such as hemoglobin and LDH from the cytosol to the extracellular space. By evaluating hemoglobin and LDH release (Figure 5A and 5B), we show no significant membrane permeabilization by any tested molecules in the range at which they have bactericidal activity in saline buffers (Figure 2A, Figure 3). This finding was confirmed by microscopic evaluation of adenocarcinoma cell morphology showing no visible difference between the control cells and those treated with 10 μg/ml LL-37, WLBU2 or CSA-13 (Figure 5C). However an increase in hemoglobin and LDH release was observed with increasing concentration. Among the three molecules tested, WLBU2 was the strongest hemolytic agent, but all of them showed similar ability to compromise adenocarcinoma cell membrane integrity (Figure 5B and 5C). CSA-13 bactericidal concentrations against *H. pylori *and *E. coli *MG1655 (Figures 2A, 2B and 3C) evaluated in saline as well as nutrient containing buffer were below its minimal hemolytic concentration and below concentrations causing dysfunction of adenocarcinoma cell membranes.

## Discussion

The rate of successful treatment of *H. pylori *stomach infection, achieved with combination therapies of two antibiotics and a proton pump inhibitor has declined from over 90% to about 80% during the past decade [27]. In addition, the cost of this therapy is significant, and therefore a need for more widely available means of treating or preventing *H. pylori *infection still exists [28]. New agents to treat *H. pylori *infections are necessary also due to increasing drug-resistance problems caused by extensive use of antibiotics [29] and the adaptive survival mechanisms of pathogenic bacteria to counteract currently used antimicrobials. For example, *H. pylori *strains resistant to amoxicillin, metronidazole and clarithromycin have been reported [30,31]. Methods to improve treatments for *H. pylori *might be guided by insight into the natural mechanisms by which infected patients respond to this bacterium and the reasons why the normal host-defense mechanisms fail.

This study confirms a previous report of increased hCAP-18/LL-37 expression in gastric mucosa of subjects infected with *H. pylori *[11]. This finding suggests that increasing production of the bactericidal peptide LL-37 may play a key role in host defense against *H. pylori *[11]. However, this bactericidal response in some subjects is insufficient and *H. pylori *infection can still reach a chronic stage. The lack of bactericidal function of LL-37 in this setting has suggested that increased expression of hCAP-18/LL-37 peptide in gastric mucus of infected subjects may have additional functions as an anti-inflammatory and growth stimulating agent. Indeed, it was recently shown that gastric ulcer healing in rats is promoted by cathelicidin-mediated transactivation of epidermal growth factor receptors (EGFR) via the transforming growth factor alpha (TGFα) signaling pathway [32]. Alternatively, loss of defense against *H. pylori *may be due to loss of antibacterial function of LL-37 in the milieu of the gastric mucosa. Consequently, design of antimicrobial agents that are more effective in this setting can be beneficial.

Motivated by immunohistological results, the activity of LL-37 against clinical isolates of *H. pylori *and *E. coli *MG1655 under biologically relevant conditions was compared with that of the synthetic peptide WLBU2 and the ceragenin CSA-13. This study shows that CSA-13, contrary to LL-37 and WLBU2 peptides, maintains strong bactericidal activity in the presence of mucin and after preincubation with pepsin at low pH. These conditions represent unique challenges related to *H. pylori *treatment, as these bacteria in the stomach are protected from the acidic environment by a thick mucus layer and the effectiveness of many antimicrobial drugs is greatly diminished at acidic pH [31]. Accordingly, the first effective therapy for *H. pylori *infection was a combination of relatively pH-insensitive antimicrobial drugs such as bismuth, tetracycline and metronidazole [33]. In addition, as the stomach periodically empties its contents (topical therapy tends to be diluted and washed out) the finding that CSA-13 has bactericidal activity at much lower concentration then LL-37, after the same incubation time (3-6 hours) [11], suggests that CSA-13 may have therapeutic potential for treatment of *H. pylori *infection. The antibacterial activity of CSA-13, which has a smaller net charge and a unique distribution of this charge over a steroid scaffold when compared with LL-37 and WLBU2 peptides, was also found to be less inhibited by mucin isolated from gastric mucosa. Therapeutic potential based on the ability of CSA-13 to eradicate *H. pylori *is also supported by previously reported antibacterial activity against other bacteria strains, including clinical isolates of *Pseudomonas aeruginosa *[21] and *S. aureus *[22]. CSA-13's unique ability to compromise bacterial membrane integrity and the chemical nature of this low-molecular-mass compound that translates to lower cost of synthesis compared to cationic antibacterial peptides suggest that CSA-13 or perhaps other ceragenins have potential for treatment of *H. pylori *infection, including those caused by its resistant strains.

## Conclusion

Bactericidal activity of ceragenin CSA-13 is maintained after preincubation in simulated gastric juice and in the presence of mucin. This *in vitro *evaluation indicates a significant potential of this molecule in treatment of stomach mucosal infection.

## Methods

### Antibacterial agents

LL-37 (NH_2_-LLGDFFRKSKEKIGKEFKRIVQRIKDFLRNLVPRTES-COOH) and WLBU2 (NH_2_-RRWVRRVRRWVRRVVRVVRRWVRR-COOH) peptides were purchased from Bachem (King of Prussia, PA). CSA-13 was prepared as previously described [34]. Amoxicillin (AMX), clarithromycin (CLR), tetracycline (TET) and metronidazole were purchased from Sigma.

### Collection of gastric mucosal and bile samples

During gastroscopy, performed with either a GIF V2 or Q145 (Olympus) gastroscope, several gastric mucosal slices were taken from the prepyloric and corpus regions of the stomach. *H. pylori *infection was established in the biopsy specimens using a urease test (CLO-test). Human bile was obtained from the gallbladder of patients undergoing cholecystectomy. Samples were filter-sterilized through a 0.45 μm membrane before being diluted in PBS (1:1) and mixed with antibacterial agents used in bacteria killing assays. The studies were approved by Medical University of Bialystok Ethics Committee for Research on Humans and Animals, and all patients gave informed written consent for participation in the study.

### Immunohistochemical studies

Immunohistochemical studies were performed on formalin-fixed, paraffin-embedded human gastric mucosal sections using a rabbit anti-LL-37 antibody (H-075-06, used at 1:100 dilution; Phoenix Pharamceuticals Inc.). Paraffin-embedded materials were sectioned to 5 μm thickness and floated on distilled water at 45°C. Sections were then mounted on slides and placed in 57°C oven overnight. The sections were deparaffinized according to standard procedures and quenched with 0.9% hydrogen peroxide in methanol for 30 minutes. The sections were incubated with primary antibody at 37°C for 60 minutes, washed with 1% PBSA (1% BSA in PBS), and subjected to binding with secondary antibody (biotinylated goat anti-Rabbit IgG, 1:400 dilution). Amplification was performed with a Vectastain ABC kit, and an HRP detection system was used to colocalize peroxidase activity with a DAB substrate. The sections were counterstained with hematoxylin. Samples were viewed with a Nikon Eclipse 80 microscope under 40× magnification.

### Evaluation of MIC and MBC

The minimal inhibitory concentration (MIC) of conventional antibiotics against seven different clinical isolates of *H. pylori *(9 × 10^8 ^CFU/ml) was determined using Muller-Hinton agar (MH) containing 5% sheep blood. The incubation was continued for 4 days at 35°C in microaerophilic conditions maintained with use of a Gas Pack-Campylobacter gas generating kit BR60. Clinical isolates of *H. pylori *were considered resistant to respective antibiotics when the MIC values were above 4 μg/ml for AMX, 1 μg/ml for CLR and 16 μg/ml for TET and Metronidazole. The minimal bactericidal concentration (MBC) of antibacterial agents was evaluated using an inoculum at 10^8 ^CFU/ml. After a 6-hour incubation at 37°C, 10 μl aliquots of the suspensions were spotted on Columbia agar supplemented with sheep blood (5%).

### Bacteria killing assay

The bactericidal activities of LL-37, WLBU2 peptides and ceragenin CSA-13 against *E. coli *MG1655 in the presence of mucin or pepsin from porcine mucus (Sigma) and human bile were measured as previously described [35]. Bacteria were grown to mid-log phase at 37°C (controlled by the evaluation of optical density at 600 nm) and resuspended in PBS buffer (pH = 7.4). The bacteria suspensions were then diluted 10 times in 100 μl of solutions containing antibacterial agents by themselves or with mucin (1000 μg/ml), or bile (the final 1:10 bile dilution mimics the environment of the upper small intestine into which bile is secreted [36] (pH = 7.4)). In another set of experiments antibacterial activity of these components was determined following their preincubation in simulated gastric juice [36,37] at pH ~1.5 with and without pepsin (0.5 mg/ml). After incubating bacteria with antibacterial molecules for one-hour at 37°C, the bacterial suspensions were placed on ice and diluted 10- to 1000- fold. Aliquots of each dilution (10 μl) were spotted on LB Agar plates for overnight culture at 37°C. The number of colonies at each dilution was counted the following morning. The colony forming units (CFU/ml) of the individual samples were determined from the dilution factor.

### Mass spectrometry

Analytical characterization was performed on the CSA-13 and LL-37 suspensions after 3H incubation with pepsin (0.5 mg/ml) at low pH (~1,5) at 37°C, using the Shimadzu (Columbia, MD) instrument (the LC-MS system consisted of a LC-20AB solvent delivery system and SIL-20A auto-sampler coupled to dual wavelength UV-Vis detector and a LCMS 2010EV single quadrupole mass spectrometer), coupled to a Shimadzu Premier C18 column (150 mm × 4.6 mm i.d., 5 μm particle size). The mobile phase flow rate was 1 ml/min with a starting ratio of 90% mobile phase A (water) and 10% mobile phase B (acetonitrile) both with 0.1% (v/v) formic acid. The analytical method consisted of the following steps: (i) sample injection and holding at 10% B for 5 min, (ii) linear gradient from 10% to 90% B over 15 minutes, (iii) holding at 90% B for 5 minutes, (iv) isocratic step to 10% B and holding for 5 minutes prior to the next sample injection. Mass spectrometry was performed on the eluent using electrospray ionization (ESI) in positive ion mode with a scanned m/z range from 160-2000.

### Red blood cell lysis

The hemolytic activity of LL-37, WLBU-2 and CSA-13 (0-200 *μ*g/ml), against human red blood cells (RBC) was tested using erythrocytes suspended in PBS. RBC prepared from fresh blood (Hematocrit ~5%) were incubated for 1 h at 37°C after addition of test molecules. Relative hemoglobin concentration in supernatants after centrifugation at 2000 × g was monitored by measuring the absorbance at 540 nm. 100% hemolysis was taken from samples in which 2% Triton X-100 was added.

### Cell culture

Human gastric adenocarcinoma cells (ATCC; CRL-1739) were maintained in DMEM (BioWhittaker) culture supplemented with 10% heat-inactivated fetal bovine serum (Hyclone) at 37°C and 5% CO_2_. For LDH release assay and microscope evaluation cells were plated in 24 well plates and grown to confluence. In all experiments, the medium was changed to serum-free media ~12 h prior to cell treatment with LL-37, WLBU2 and CSA-13 (0-200 μg/ml) in individual wells, for 1 hour. Cell culture medium was then collected, centrifuged (10 mins, 5000 rpm, RT) and subjected to LDH evaluation (LDH-cytotoxicity Assay Kit; BioVision Inc.)

## Competing interests

Dr P. Savage is a paid consultant for Ceragenix Pharmaceuticals, Innate Immune Inc., and WittyCell. None of the research reported in this paper was supported by Ceragenix Pharmaceuticals or by any other corporate entity. Other authors: none to declare.

## Authors' contributions

KL: carried out the *H. pylori *study, bacteria killing assay, performed the statistical analysis and drafted the manuscript; AN: carried out immunohistochemical studies; DF: carried out mass spectrometry; QW: participated in the *H. pylori *study; ZN: collection of gastric mucosal and bile samples, participated in the design of the study and drafted the manuscript; PS: carried out CSA-13 synthesis and participated in study design and helped to draft the manuscript; SD: involved in mass spectrometry analysis and helped to draft manuscript; PJ: participated in study design and helped to draft the manuscript; RB: carried out red blood cell lysis and cell culture study, participated in study design and helped to draft the manuscript. All authors read and approved the final manuscript.
